# Supplementary material for: Potential links between human bloodstream infection by Salmonella enterica serovar Typhimurium and international transmission to Colombia
Source: PLoS Negl Trop Dis. 2025 Jan 28;19(1):e0012801. doi: 10.1371/journal.pntd.0012801 (PMC11790238; doi:10.1371/journal.pntd.0012801)
Supplement: S1 Fig — The coloured rings show the serotyping from different methods. Branches with bootstrap values less than 80 were marked in grey. (PDF) [file pntd.0012801.s004.pdf]

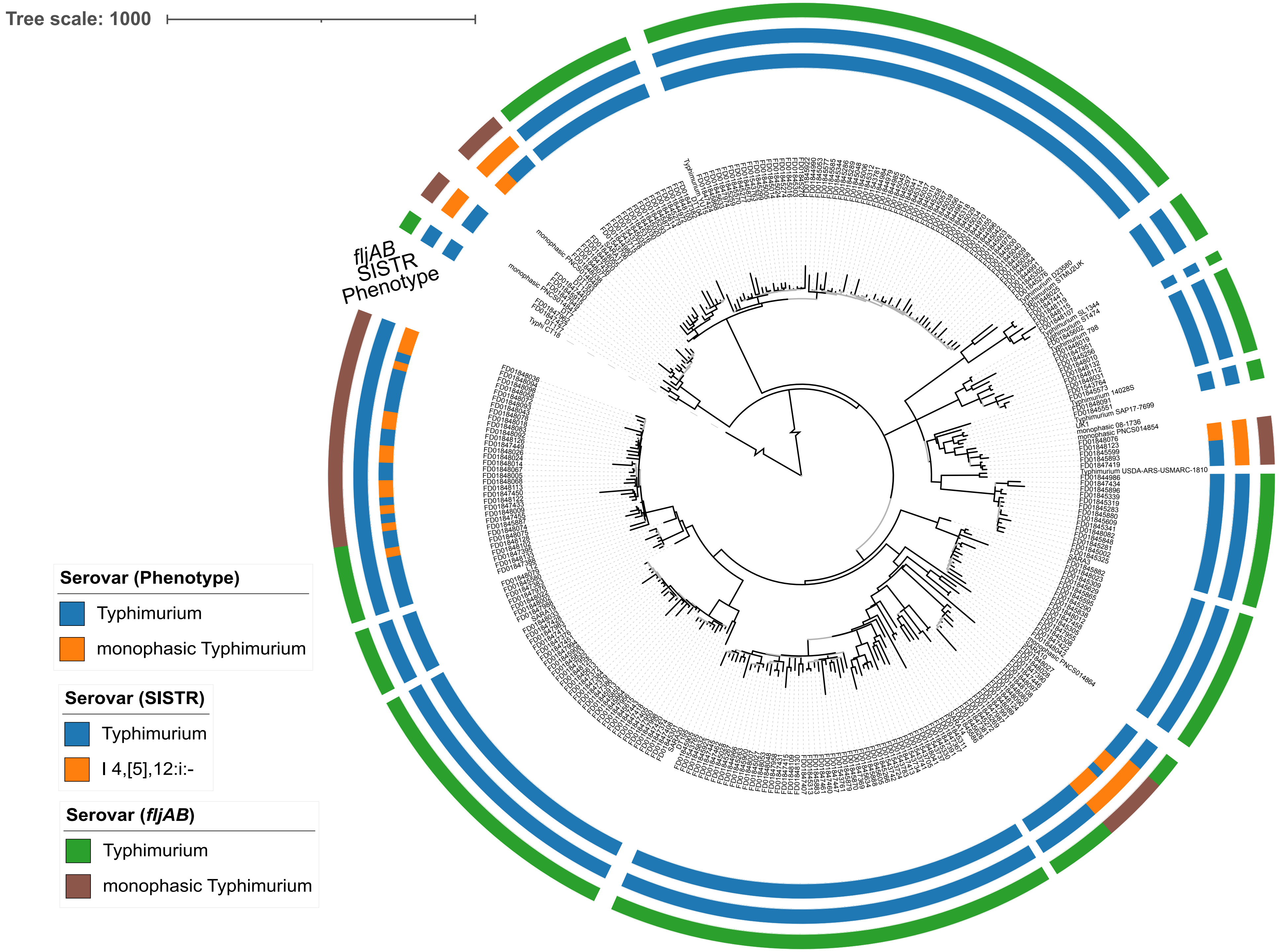

S4 Figure: The detailed Raxml tree showing monophasic Typhimurium isolates identified from 3 different methods: experimental serovar identification, the in-silico prediction by SISTR, and the presence or absence of the fljAB genes. The coloured rings shows the serotyping from different methods. Branches with bootstrap values less than 80 were marked in grey.
