# Supplementary material for: Potential links between human bloodstream infection by Salmonella enterica serovar Typhimurium and international transmission to Colombia
Source: PLoS Negl Trop Dis. 2025 Jan 28;19(1):e0012801. doi: 10.1371/journal.pntd.0012801 (PMC11790238; doi:10.1371/journal.pntd.0012801)
Supplement: Attachment — Submitted filename: Rebuttal_letter - PLoS NTD -FINAL [NoTkCh].docx [file pntd.0012801.s005.docx]

### Response to Reviewer’s Comments

The authors responses are in Blue font below.

### 1. Methods

-Are the objectives of the study clearly articulated with a clear testable hypothesis stated?</br> -Is the study design appropriate to address the stated objectives?</br> -Is the population clearly described and appropriate for the hypothesis being tested?</br> -Is the sample size sufficient to ensure adequate power to address the hypothesis being tested?</br> -Were correct statistical analysis used to support conclusions?</br> -Are there concerns about ethical or regulatory requirements being met?</br></br>

**Reviewer #1**: See below for the specific points

Reviewer #2: The methods are clearly written with the study design appropriate. The authors have undertaken multiple different analyses. There were a few areas where additional clarity would be useful

-	I have no concerns about the ethics but I couldn’t find a statement. I would assume these fall under the 10,000 genome project and/or were isolates collected under public health acts / in the public domain. The authors could include a sentence to address this.

Thank you for posing this important question. We have added a declaration that the ethics approval does not apply to this study because the genome sequences had been published as part of the 10,000 *Salmonella* genomes project (Perez-Sepulveda *et al.,* 2021 [<https://genomebiology.biomedcentral.com/articles/10.1186/s13059-021-02536-3> ]).

-	For the AMR genotype phenotype concordance analyses with the authors reporting phenotypes for multiple drug classes. However, there doesn’t seem to be statistical analysis e.g of the sensitivity / specificity of the AMR genotype phenotype profiles. From Fig 3 it looks to have high concordance but this was from visually look at the heatmap.

We agree that a statistical analysis is the better way to show the AMR genotype phenotype concordance than the visualization in a heatmap. Accordingly, we calculated the sensitivity/ specificity of the genotype prediction and added the % of true positives/ true negatives in the main text and in Sup Table S1.

The observed phenotypes demonstrated high concordance with the genotypic profiles. For example, resistance to ampicillin was associated with the presence of resistance genes including *blaCARB-2, blaCMY-2, bla-CTX-M-12, blaCTX-M-15, blaOXA-1, blaOXA-2, blaOXA-392, and blaTEM-1B*. Predictions of ampicillin resistance based on the presence of these genes exhibited a sensitivity of 89.2% and a specificity of 95.2% (S1 Table).

-	ML trees – these were inferred from SNPs in core gene alignments from Panaroo. Was the default flag for core used in Panaroo and what tool was used for the alignment? MAFT? Were the core gene numbers similar / different between the different trees inferred?

Yes, the default flag for core was used in Panaroo. The core gene alignment is generated in Panaroo itself. The core gene numbers are different between the various trees, based on the genomic similarity of the isolates in the individual tree.

In the tree of 270 Colombian isolates and 22 contextual isolates, Panaroo generated the core genome alignment from 4170 core genes. Furthermore, the core genome alignments of HC100_305, HC100_20801, and HC50_64733 clusters were generated by Panaroo from 4463, 4456, and 4311 core genes, respectively.

-	Identify circularised contigs from draft assemblies. This was important for the subsequent plasmid analyses. Do you mean a single contig that circularised? Yes. Or several contigs that were fell out in the assembly graph? Additionally, I think there should be an additional cite for plsdb (<https://ccb-microbe.cs.uni-saarland.de/plsdb/>). I hadn’t heard of this tool before and wondered what the difference was compared to e.g. Mob-suite?

Thank you for suggesting the additional citation for PLSDB. We have added the link to the methods section. PLSDB is a manually curated database for identifying previously characterized plasmids. Queries for the circularized contigs to similar plasmids in PLSDB are performed using BLASTn. PLSDB differs from MOB-suite, which is a software toolkit designed for typing plasmids from whole-genome sequencing data.

-	HeirCC was important in to this story but there was limited explanation of how the scheme in enterobase works. E.g that is cgMLST based and what the different HC levels mean (HC100 v HC50). Also how the levels relate to STs. E.g. HC100_20801 was the ST313 with sublineages in it?

HierCC is a system that classifies bacterial genomes into different levels of clusters based on the cgMLST allele distances. For example, the genomes within the same HC100 cluster have fewer than 100 allele differences. The HC5 or HC10 categories are usually used to identify outbreaks; HC50 and HC100 are usually used to identify lineages. The Colombian S. Typhimurium clusters were defined at the HC100 level. For Cluster 7, three sub-clusters were identified at the HC50 level. The transmission history of three Colombian clusters was traced in this study, including cluster 2, cluster 3 and sub-cluster 7.3, which belong to HierCC clusters HC100_20801, HC100_305 and HC50_64733, respectively. The *Salmonella* genomes that belonged to the same clusters from the other countries were queried via Enterobase, as stated in the text.

For the second question, STs are generally categorised to the HC900 level. HC100_20801 is a sublineage of ST313 isolates. The HC100_20801 cluster included the Colombian ST313 isolates, the UK-ST313, the Malawian ST313 Lineage 3, and other ST313 isolates from Africa, North America and South America.

-	In both methods and results, the authors used different names for the clusters (HierCC, cluster and ST). At times I had keep referring back to Fig 1 to work out what cluster was being discussed.

We are grateful for this helpful suggestion, and realise that the use of different names for clusters added complexity for the reader. To clarify this issue, we assigned the cluster numbers Cluster 1–7 for the Colombian isolates, which are now used throughout the text for reader convenience. The corresponding HierCC cluster IDs are also included in the figures to add important context of relevance to public databases. In most cases, the terms Cluster 1–7 was sufficient for understanding the results. When discussing global transmission, it was necessary to reference the HierCC IDs of genomes from EnteroBase to provide context. Additionally, names like DT104 and ST313 were used to incorporate context with previous publications.

-	Bactdating – default parameters in the R package?

Yes, we used the default Bactdating parameters.

Reviewer #3: (No Response)

--------------------

2. Results

-Does the analysis presented match the analysis plan?</br> -Are the results clearly and completely presented?</br> -Are the figures (Tables, Images) of sufficient quality for clarity?</br></br>

**Reviewer #1:** the results presented in a well-standard form.

**Reviewer #2:** The results matched the analysis plan with the results clearly presented. There were a few things I think could be expanded upon that would strengthen the manuscript

-	The 20 unknown STs – several looked to be part of HierCC_100_305. Were these SLV of the main ST of this group?

MLST (<https://github.com/tseemann/mlst>) predict STs from 7 housekeeping genes. The STs of 20 isolates were unknown because at least one of the seven housekeeping genes used for the MLST analysis was incomplete following genome assembly.

-	For the AMR results, there were a few isolates that had 3GCs resistance detected and I think were phenotypically resistant as well. Were these in the same lineages/ clusters or spread across the population?

I think it is important to report on these mechanisms even if at low frequency in the dataset, as it could be the detection of emerging resistance to this important drug class in Latin America (also a priority for the WHO). Related – please cite the WHO Bacterial Priority List for 2024 instead of 2017. I don’t think colistin or carbapenem resistance was detected in the Colombian dataset. Again I think it is useful to note the absence of AMR mechanisms to these drug classes as well.

We are grateful for the insightful suggestions regarding the AMR results. We have updated the citation of the WHO Bacterial Priority List, which specifies that fluoroquinolone-resistant *Salmonella* is now a high priority pathogen.

As well as the clade-specific antimicrobial resistance mentioned above, sporadic isolates exhibited resistance to third-generation cephalosporins (3GCs), including ceftazidime and cefotaxime. This resistance was likely conferred by the Extended-Spectrum Beta-Lactamase (ESBL) genes *blaCMY-2, blaCTX-M-12*, and *blaCTX-M-15*. The emergence of 3GC-resistant isolates is noteworthy, particularly because 3GC-resistant Enterobacterales are classified as a critical priority on the WHO Bacterial Priority Pathogens List. Fortunately, carbapenem resistance, another critical priority of the WHO Bacterial Priority Pathogens List, was not detected in the Colombian S. Typhimurium dataset.

-	In reporting the point mutations in QRDR regions associated with quinolone resistance, please report the specific mutations (page 15 and supp table quinolone tab), especially as combinations of these point mutations are associated with different phenotypes (e.g. 1 point vs 3). Were any point mutations in parC codon 80 detected? No

Thank you for the suggestion. We have reported the specific *gyrA* mutations (GyrA p.S83F or p.D87Y) in the text and included the details in Supp table 1.

-	Plasmid presence – Fig 3. The heatmap shows plasmid cov including for the pCOT7 which looks to have ~0.4 coverage. I couldn’t see the details of this plasmid in Supp table 2 reference plasmid tab – I was looking as I wanted to know the plasmid length. Would the authors consider it important to have a threshold for the plasmids in this analysis? E.g > 0.8 coverage of plasmid to have confidence in that plasmid (or something close) in the population. In Fig 3 – it was difficult to distinguish between 0.8 and 1 coverage. Suggest changing the colours to make this easier to interpret.

We agree that it is important to have a threshold for the plasmids in the Colombian population. We have revised Figure 3 to show only the plasmids with >0.8 coverage.

-	There was some text in the results that I think belong in the methods. E.g. first para of page 18 “To identify the non-pSLT..”

We thank the reviewer for pointing this out. This paragraph is a repetition from the methods. It has been deleted and the rationale has been summarised in a single sentence. The non-pSLT plasmids were extracted from the single circular contigs assembled from the Illumina reads.

-	Numbers for the different clusters. In the text HC100_20801 has 87 isolates but the Supp Table 3 had 95 isolates- why was there a difference?

We thank the reviewer for carefully checking the data. The 87 “ contextual isolates” were the genomes downloaded from Enterobase and used in the study as a reference, in addition to the 5 Colombian Cluster 3 isolates that we identified. The number of genomes used in the tree is 92. However, three isolates in Supp Table 3 were repeated because the genomes were uploaded to Enterobase twice. These 3 genomes have been deleted from Supp Table 3.

-	Some text in results would be better in the discussion / conclusions. E.g last para of page 23 discussing the lack of animal models.

We agree that the last paragraph of page 23 was redundant component of the Results section. It has now been cut to 3 sentences that explain the rationale of using the invasive index, shown below:

This study focused on the genomes of S. Typhimurium isolates that cause bloodstream infection. However, the lack of animal models that faithfully recapitulate human invasive infection has prevented meaningful experimental comparisons between the bloodstream and gastrointestinal isolates in the context of iNTS disease. Therefore, a machine learning-based prediction, Wheeler’s invasiveness index, was used to compare the invasive potential of S. Typhimurium from each HC100 cluster.

-	For the invasive index analyses – it was hard to determine the significance of the index in the population. There were differences but from Fig 6, all values were <0.20 but in the previous para on page 24 the authors state that a high index is a signal these bacteria are adapting to extraintestinal lifestyle. Do the authors know how e.g. Typhi performs with this index? Is 0.1 high?

We thank the reviewer for this useful suggestion. We compared the invasiveness index to the African iNTS-associated ST313 lineages, for which the invasive potential has been studied previously. Specifically, Typhi has an index of >0.8, while the African iNTS-associated Enteritidis has an average index of about 0.25, and African iNTS-associated Typhimurium ST313 is around 0.15-0.20. We have now updated Figure 6 to provide a more thorough comparison of the Colombian Typhimurium to these invasive lineages.

Reviewer #3: (No Response)

--------------------

3. Conclusions

-Are the conclusions supported by the data presented?</br> -Are the limitations of analysis clearly described?</br> -Do the authors discuss how these data can be helpful to advance our understanding of the topic under study?</br> -Is public health relevance addressed?</br></br>

Reviewer #1: see the specific points.

**Reviewer #2:** The conclusions are largely supported by the results. Additional clarification of methods and results will help. The public health relevance was clearly addressed.

-	Analysis on HIV infection and trying to explore the host factors was reported in the discussion / conclusion (page27). Should this be included in methods and results? Or was the point that it was difficult to explore host factors.

The lack of available patient data makes it difficult to prove the association of the host factors with *Salmonella* genomic differences. The mention of HIV infections represents an inference rather than a more solid analysis. Accordingly, this point is made in the Discussion sction of the manuscript.

-	The food exports section is interesting but it is possible it could be the movement of people that then spread the bacteria to livestock. Suggest rephrasing this text a bit.

We agree that the transmission may also be driven by the movement of other food products or human travel, and have rephrased the text accordingly.

-	A paragraph on the strengths and limitations of this study would be valuable. For example, a key strength is the dataset from a region where to date there has been a paucity of data (e.g. in the recent Carey 2023 eLIFE on Typhi there was limited isolates from Latin America). A limitation would be the sampling biases in the public data and some consideration as to how this may impact findings based on sampling approaches).

We thank the reviewer for the insightful comment. We agree that a paragraph on the strengths and limitations of this study is valuable.

However, it is important to note that the genomic data present in Enterobase suffers from significant sample bias, with a skew towards genomes originating from gastroenteritis-associated Salmonella in high-income countries (particularly in the EU and North Americ). Our study helps to bridge this gap by incorporating genomic data obtained through systematic surveillance in Colombia, providing valuable insights into Salmonella Typhimurium within Latin America. As our research focused on human bloodstream-derived isolates rather than stool or environmental samples, its broader applicability to public health strategies in Colombia is limited.

Reviewer #3: (No Response)

--------------------

4. Editorial and Data Presentation Modifications

Use this section for editorial suggestions as well as relatively minor modifications of existing data that would enhance clarity. If the only modifications needed are minor and/or editorial, you may wish to recommend “Minor Revision” or “Accept”.

**Reviewer #1:** all the new data should be free in the public domain.

We have requested that NCBI release our data with immediate effect.

**Reviewer #2: -**	The introduction had only 8 references and some of these were dated. E.g. an important genomic study of monophasic salmonella was the Petrovska 2016 EID and this wasn’t cited until the discussion. There was a bias in references to those from the UK. Additional references eg from Europe, North America for implementation of routine WGS and use of genomics to explore typhimurium and monophasic in settings in Africa and Asia (e.g Van Puyvelde 2023 Nat Comms for iNTS in sub Saharan Africa and The 2023 Comms Bio for MDR plasmids in South East Asia) would provide greater context for this study especially given the focus on global spread of the pathogen

-	Define MDR in the introduction – it is explained in the results

-	Explain what iNTS is and what is meant by ‘systematic iNTS disease’. This is really important to the underlying rationale for this study

-	The authors refer to DT104 throughout the manuscript. I think this relates to phage typing that used to be done for Salmonella? Some explanation of changes to surveillance efforts for NTS and how genomics has changed it etc would be really useful in the introduction. This would help the broad readership of PLoS NTD know what DT104 means and why it is important (or has this nomenclature been replaced?)

We are grateful for the reviewer’s thoughtful suggestion for improving the Introduction. We have now added text to define the term “MDR” in the introduction, and have cited recent relevant Typhimurium WGS studies. We have also added more background on iNTS and DT104 as follows:

Although usually associated with self-limiting diarrhoea, non-typhoidal *Salmonella* serovars can also spread from the human intestine to normally sterile sites and develop bacteraemia, meningitis, and other focal infections, collectively referred to as Invasive Non-Typhoidal *Salmonella* (iNTS) Disease [8]. In 2017, fatal iNTS bloodstream infections were responsible for more than 77,000 deaths worldwide[9]. In sub-Saharan Africa (SSA), iNTS disease has been associated with two distinct lineages of *S.* Typhimurium ST313, both carrying MDR-encoding Tn21 elements on plasmid pSLT [10] and the prophages, BTP1 and BTP5 [11]. A third pan-susceptible lineage of S. Typhimurium ST313 was found in Malawi recently, with a higher invasiveness index [12]. ST313 Lineage 3 was closely related to ST313 isolates responsible for gastroenteritis in the UK and Brazil [13, 14]. However, while *S*. Typhimurium ST313 is known to be a major cause of iNTS disease in Africa [10, 12], the role of ST313 in Colombia has been unclear.

The Multidrug-resistant *S*. Typhimurium phage type DT104 emerged in the 1980s, and subsequently became the dominant *Salmonella* clone that is associated with infection in both agricultural animals and humans in European and North American countries [15]. DT104 isolates were resistant to ampicillin, chloramphenicol, streptomycin, sulphonamides and tetracyclines (ACSSuT resistance type) [16]. A WGS-based analysis revealed that DT104 initially acquired MDR determinants and was subsequently transmitted to American countries such as the US, Canada, and Argentina via separate routes [17]. Investigation of DT104 isolates sampled from the human bloodstream and stool demonstrated that DT104 was no more invasive than other common *Salmonella* serovars [18].

Reviewer #3: (No Response)

--------------------

5. Summary and General Comments

Use this section to provide overall comments, discuss strengths/weaknesses of the study, novelty, significance, general execution and scholarship. You may also include additional comments for the author, including concerns about dual publication, research ethics, or publication ethics. If requesting major revision, please articulate the new experiments that are needed.

**Reviewer #1**: The study provides valuable insights into the genomic characteristics of Salmonella Typhimurium isolates responsible for bloodstream infections in Colombia, particularly emphasizing the identification of ST19 as the predominant sequence type. The investigation into the potential international transmission and the comparison with global isolates adds significance to the findings. However, the novelty of the study could be better emphasized by providing a deeper discussion on how these findings could influence public health strategies in Colombia and similar regions.

Thank you for the reviewer’s thoughtful comments. While we agree that our study provides valuable insights into the genomic characteristics of *Salmonella* Typhimurium isolates and their potential public health implications, we respectfully clarify that the direct influence of public health strategies was not the primary aim of this research. As researchers from a different country, we believe it would be presumptuous to make direct recommendations regarding public health strategies in Colombia. Instead, we hope our findings will serve as a foundation for further studies, and will serve as the basis for discussions led by local experts and public health authorities.

**Major points**

Phylogenetic Analysis: The phylogenetic analysis appears robust, linking Colombian isolates to global clusters. However, the manuscript would benefit from a more detailed explanation of the methods used for phylogenetic tree construction and statistical support (e.g., bootstrap values). Additionally, while the study identifies multiple independent transmission events, it would be helpful to discuss potential sources or routes of these transmissions in more detail.

We thank the reviewer for the comment, the bootstrap values are now detailed in the Methods and the Results. The maximum-likelihood phylogenetic tree was inferred with Raxml-ng v0.6.0 [43] using the GTR+G model from the core genome alignment with 100 bootstrap iterations. The phylogenetic tree demonstrated robust support for the major clades (bootstrap > 80%).

We have discussed the potential source of the transmission of Sub-cluster 7.3. However, the link to food animals remains very speculative. Accordingly, we have avoided overstating the importance of our findings.

Plasmid and Antimicrobial Resistance (AMR) Analysis: The study highlights the presence of MDR profiles and plasmid-associated resistance. While this is well documented, the manuscript could provide more information on the functional impact of these plasmids. For instance, how do these plasmids contribute to the fitness of S. Typhimurium in the host environment? Are there any potential targets for novel therapeutics identified in these plasmids?

Thank you for these insightful comments. A detailed discussion of the functional impact of these plasmids is beyond the scope of this study, which did not involve specific investigations of plasmid biology. Consequently, it remains unclear how these plasmids contribute to the fitness of *S.* Typhimurium in the host environment. To our knowledge, studies exploring this aspect in this context have not yet been conducted.

We have, however, reported the AMR genes carried by the plasmids, which may prove valuable for clinical epidemiologists and could inform future research. Unfortunately, it is not currently possible to identify potential targets for novel therapeutics from these plasmids based on the data presented in this study.

ST313 in Colombia: The identification of ST313 in Colombia, a sequence type predominantly associated with sub-Saharan Africa, is a crucial finding. The study discusses its limited prevalence in Colombia, which is attributed to the immune-competence of the population. However, this assertion could be further supported by epidemiological data or references to similar studies in other regions with immune-competent populations. Moreover, it would be valuable to discuss the potential public health implications if ST313 were to become more prevalent.

Thank you for this thoughtful comment. We have added a comparison of the limited prevalence of ST313 in Colombia to the current situation in Asia.

We speculate that the high incidence of iNTS and the prevalence of S. Typhimurium ST313 in sub-Saharan Africa (SSA) reflects a combination of two factors that are absent from Colombia: the genetic adaptation of S. Typhimurium ST313 lineage 2 to the extraintestinal lifestyle and the vulnerability of the human population. A similar situation can be found in Southeast Asia and South Asia, where the incidence of iNTS and HIV-associated cases is significantly lower than in SSA [9], and the proportion of ST313 isolates is limited [71].

Data Interpretation and Public Health Implications: The manuscript could expand on the public health implications of these findings. How can this genomic information be used to enhance current surveillance systems in Colombia? Are there specific recommendations for public health policy that could arise from this study, such as the monitoring of particular MDR strains or the implementation of targeted interventions?

The majority of S. Typhimurium isolates in the DT104-associated Cluster 2 exhibited resistance to ampicillin, streptomycin, tetracycline, and sulphonamide. Certain isolates from Cluster 2 also demonstrated resistance to chloramphenicol, trimethoprim, or azithromycin **(Fig 2)**. Subsequently, after 2010, Sub-cluster 7.3 acquired quinolone resistance encoded by *qnrB19*.

The replacement of Cluster 2 by Cluster 7 in around 2010 was associated with the acquisition of quinolone resistance and a reduced incidence of ampicillin resistance. The rise of fluoroquinolone-resistant Sub-cluster 7.3 warrants close monitoring by Colombia’s public health departments.

Comparative Analysis with Global Isolates: The manuscript does a good job of placing the Colombian isolates in a global context. However, the discussion could be strengthened by providing more comparative insights between the Colombian isolates and those from other regions, particularly in terms of genomic variations and their potential functional consequences.

We speculate that the lower incidence of iNTS and the limited prevalence of S. Typhimurium ST313 in Colombia compared to in SSA reflects a combination of two different factors: the genetic adaptation of S. Typhimurium ST313 to the extraintestinal lifestyle and the relatively immunocompetent status of the Colombia population.

A similar situation can be found in Southeast Asia and South Asia, where the incidence of iNTS and HIV-associated cases is significantly lower than in SSA [9], and the proportion of ST313 isolates is limited [71].

**Minor Points**

The methods section, while comprehensive, could benefit from a clearer description of the bioinformatic tools and parameters used. For example, the criteria for defining clusters and the thresholds for AMR gene identification should be explicitly stated.

Figures and Tables:

The figures and tables are informative but could be more effectively used to highlight key findings. For instance, a figure summarizing the phylogenetic relationships alongside plasmid and AMR profiles would provide a visual overview of the study's main points.

We consider that this type of visual overview is not essential as Figures 2 and 3 already show AMR profiles and plasmid profiles in the context of an identical phylogenetic tree. We hope this will be acceptable to the editor.

Additionally, more detailed legends explaining the figures would help readers interpret the data more easily.

We agree that the original versions of the figure legends lacked some of the detail needed to interpret the data. Accordingly, we have rephrased the legends of figures 1, 2, 3, 4 & 5.

While the manuscript is well-referenced, it could incorporate more recent studies on Salmonella genomics, particularly those focusing on Latin American countries. This would help place the findings in a broader context and demonstrate how they advance the current understanding of Salmonella epidemiology.

Thank you for this valuable suggestion. We have added references to recent studies from Africa, Southeast Asia and South Asia to contextualise the study and compare the Colombian data.

The manuscript is generally well-written, but some sentences could be revised for clarity. For instance, the explanation of the significance of ST19 versus ST313 in the Colombian context could be more concise and directly linked to the study’s conclusions.

We have revised the sentence to clarify the significance of ST19 versus ST313 in Colombia. We speculate that the lower incidence of iNTS and the limited prevalence of *S.* Typhimurium ST313 in Colombia compared to in SSA reflects a combination of both pathogen and host factors: the genetic adaptation of *S.* Typhimurium ST313 to the extraintestinal lifestyle in vulnerable patients and the immunocompetent status of the Colombian population.

These comments should provide a balanced perspective on the manuscript, acknowledging its strengths while offering constructive suggestions for improvement. If further detailed analysis or additional specific critiques are needed, please let me know.

**Reviewer #2**: Overall this was an interesting and well-presented study on a topical area. The new sequence data in this study will be of use to researchers global given the limited data on Latin America to date.

**Data availability**

-	The authors have made a very good effort at making the data available in this study. In the data availability statement it says the accessions are available in S1 table. I could see the barcode and strain name in the Main tab but not the accession. Please add the SRR/ERR for each isolate.

We have added the SRR/ERR for each isolate in S1 table.

-	I looked for PRJNA1095721 but couldn’t find this BioProject. Please make public. It was unclear if the complete genome sequences of the novel plasmids were from the short read data reported in this study. Or if ONT data was used. Please make this clear and if ONT data was used – additional methods need to be included.

We have requested that NCBI release our data with immediate effect.

**Reviewer #3:** In this study, Li et al report and analyze genomic characteristics of a library of S. Typhimurium strains isolated from individual with bloodstream infections in Colombia. Whole genome sequencing for 270 strains is performed, and the phylogeny of these strains (including various reference strains) was performed. Resistance to various antimicrobials is predicted from the genome assemblies, and confirmed phenotypically. Plasmid content and distribution is analyzed as well. The most interesting part of the study is the attempt at retracing global transmission routes of strains isolated from Colombia. Cluster 2 strains (MDR DT104-related) were apparently introduced at least three different times from different sources. Quinolone/chloramphenicol resistant strains originated in Colombia and were introduced to other countries. Cluster 3 (invasive NTS-associated ST313, originally identified in Africa) was introduced to Colombia through at least two distinct events.

Invasiveness of strains was predicted based on genomic content.

Overall, this study addresses an important need. Most studies on the genomics of Salmonella (invasive and gastroenteritis-associated strains) are done on isolates from Europe and the US, due to sampling bias. In this study, the authors focused on Colombian isolates, which is a strength of this study. The most interesting finding is the (apparently) frequent exchange of S. Typhimurium isolates with other countries, as shown in Fig. 4 and 5. Main conclusions are justified, the reading is clear, and the data are presented well. I have no major concerns. Some minor comments for the authors’ consideration are below.

Minor comments, edits:

There are a lot of abbreviations used in the main sections. Some of them are unavoidable, such as strain designations, etc. To improve clarity, I would suggest to keep all other abbreviations to a minimum. For example, SSA (sub-Saharan Africa), iNTS, AMR, MDR.

Thanks for the suggestion. For the abbreviations used only one or two times, such as Instituto Nacional de Salud (INS), Minimum Inhibitory Concentration (MIC), low- and middle-income countries (LIMCs), we have now used full names instead.

Pge 27: “Here. We investigated”

We have corrected accordingly.
